# Assessment of Potential Nitrite Safety Risk of Leafy Vegetables after Domestic Cooking

**DOI:** 10.3390/foods10122953

**Published:** 2021-12-01

**Authors:** Songheng Wu, Yuhuan Liu, Xian Cui, Qi Zhang, Yunpu Wang, Leipeng Cao, Xuan Luo, Jianghua Xiong, Roger Ruan

**Affiliations:** 1 Engineering Research Center for Biomass Conversion, Ministry of Education, State Key Laboratory of Food Science and Technology, College of Food Science and Technology, Nanchang University, Nanchang 330047, China; wsh_magnus@163.com (S.W.); cuixian@ncu.edu.cn (X.C.); zhangqi09300218@163.com (Q.Z.); wangyunpu@ncu.edu.cn (Y.W.); caoleipeng2@163.com (L.C.); ncuspyluoxuan@163.com (X.L.); 2 Agricultural Ecology and Resources Protection Station of Jiangxi Province, Nanchang 330046, China; jxnyb@126.com; 3 Center for Biorefining and Department of Bioproducts and Biosystems Engineering, University of Minnesota, Paul, MN 55108, USA; ruanx001@umn.edu

**Keywords:** cooking, leafy vegetable, nitrate, total antioxidant capacity, antioxidant/in vivo nitrite ratio, potential nitrite safety risk assessment

## Abstract

Improper cultivation can easily cause excessive nitrate accumulation in leafy vegetables, and the cooking processes used to prepare them can upset their nitrate/antioxidant balance, affecting their potential nitrite safety risk (PNSR). We investigated the impacts stir-frying, steaming, microwaving, and boiling on the nitrate, nitrite, and antioxidant capacity in water spinach and cabbage, and observed the impacts of storage duration on the PNSR. The antioxidant/in vivo nitrite ratio (A/N) was used to evaluate the nitrite risks in the cooked vegetables. Boiling achieved the highest A/N ratio (1.57) for water spinach, reducing the nitrate content by 25% without significantly affecting the antioxidant capacity. Stir-frying achieved the highest A/N ratio (6.55) for cabbage, increasing the antioxidant capacity by 140% without significantly affecting the nitrate content. Furthermore, it was found that the storage periods for boiled water spinach and stir-fried cabbage should not exceed 12 h and 24 h, respectively. Appropriate cooking methods and limited storage times are thus required for leafy vegetable to prevent adverse health effects.

## 1. Introduction

Leafy vegetables are an important part of the human diet due to their abundant vitamins and fibers. However, due to the abuse of chemical fertilizers and unreasonable planting methods, the nitrate content of intensively planted vegetables tends to reach excessively high levels [1]. Approximately 80% of total nitrate ingestion has been reported to come from vegetables, and leafy vegetables easily accumulate nitrate [2]. According to Khan et al. [3], approximately 6–7% of the total ingested nitrate is converted to nitrite in the mouth under the action of oral bacteria. In addition, nitrates can easily be transformed into nitrites or other N-nitroso compounds owing to improper handling during storage. Nitrites are considered a health risk factor since they can combine with amines in the presence of stomach acid to form N-nitrosamines, which are highly carcinogenic [4]. Nitrites are also believed to be associated with methemoglobinemia syndrome, colloquially referred to as ‘blue baby syndrome’ [5]. Therefore, excessive nitrate intake is associated with potential health risks.

Nevertheless, leafy vegetables contain several antioxidant substances, such as ascorbic acid, polyphenols, and carotenoids, which can eliminate nitrites and block the synthesis of N-nitrosamines [4]. Ascorbic acid is the main antioxidant in gastric juice, and it exerts a protective effect against gastric cancer. It exhibits a strong nitrite-scavenging ability by directly reducing nitrite to nitric oxide. A natural derivative of vitamin C (L-ascorbic acid) isolated from goji berries (*Lycium barbarum* L.) also showed nitrite-scavenging ability [6]. Polyphenols are a main source of antioxidant activity in plants. The hydroxyl group of phenolic compounds can inhibit the formation of N-nitrosodimethylamine, and nitrite can be reduced to nitric oxide by phenols at low pH [7]. Polyphenol extracts from plants, such as guava [8] and apple [9], have been reported to have the ability to remove nitrite and block the synthesis of N-nitrosamines. Although endogenous nitrite is at risk of being converted to nitrosamines, under the action of antioxidants, nitrite is reduced to nitric oxide and the formation of nitrosamines is inhibited. In a previous study using an in vitro model system, when the molar ratio of ascorbic acid to nitrite was greater than 2:1, the formation of nitrosamines was completely blocked [10]. Therefore, the potential nitrite safety risk (PNSR) in leafy vegetables depends on the balance between antioxidants and nitrate. The antioxidant/in vivo nitrite ratio (A/N) has been used to assess whether the nitrite or nitrate content in pickled fruit and vegetable products could be considered harmful to human health in a previous study [10].

Various forms of heat treatment, which are common cooking methods in many homes, can change the structure and properties of food, kill harmful microorganisms, and render food more consumable, nutritious, and safe. Cooking can change the composition of vegetables. However, antioxidants and nitrates are inconsistently affected by the cooking process, which can upset the balance between them and increase the PNSR of leafy vegetables [11,12]. Huarte-Mendicoa et al. [13] found that the nitrate content of broccoli was reduced by 22–79% after boiling, while Prasad and Chetty [14] found that the nitrate content of fresh leafy vegetables fried in soybean oil increased by 159–307%. Turkmen et al. [15] found that boiling decreased the antioxidant activity of peas and leek but increased the antioxidant activity of spinach, broccoli, green beans, squash, and pepper. Similarly, Wachtel-Galor et al. [16] found that microwaving reduced the antioxidant capacity of cabbage and choy-sum by 50% and 30%, respectively, but increased the antioxidant capacity of cauliflower and broccoli by 75% and 40%, respectively. At present, most studies focus on evaluating the effect of cooking method on individual antioxidant compounds or nitrates; fewer studies have reported on their balance in vegetables and their combined contribution to PNSR of cooked vegetables.

In this study, we used two commercially available leafy vegetables (water spinach and cabbage) as samples to explore the effects of four common domestic cooking treatments (stir-frying, steaming, microwaving, and boiling) on nitrate, nitrite, and antioxidant capacity. Subsequently, the potential nitrite safety risk of cooked vegetables was evaluated on the basis of the A/N ratio reflecting the balance between antioxidant capacity and nitrate, which has not been reported in previous studies. In addition, when cooked vegetables are not eaten in time, their nitrite safety risk may be changed during storage under the action of microorganisms. Therefore, we additionally explored the actual nitrite safety risk following overnight storage of vegetables cooked by suitable methods.

## 2. Materials and Methods

### 2.1. Materials

Water spinach (*Ipomoea aquatica* Forssk.) and cabbage (*Brassica oleracea* L.), commonly consumed in China, were purchased from a local market in Nanchang, China. These vegetables were sorted (weeds and rotting leaves were removed), washed, drained, and finally cut into similar-sized pieces and thoroughly mixed. Sunflower seed oil for stir-frying was purchased from a local supermarket. The data for raw vegetables and sunflower seed oil are listed in Table 1. Since the antioxidant property of sunflower seed oil is almost negligible, it is not believed to affect the experiment (Table 1).

### 2.2. Cooking Process

Water spinach and cabbage were subjected to four common domestic cooking methods: stir-frying, steaming, microwaving, and boiling. Vegetables were divided into five equal portions by weight; one portion was kept raw, and the other four were used for cooking. Four repetitions were set for each treatment. During the cooking process, the high temperature of stir-frying and microwaving causes the vegetables to lose water quickly and appear as blackened parts (Figure S1). To ensure the edibility and uniform processing times, the cooking time was set to 2 min by considering the color, smell, and taste of the vegetables.

Stir-frying: sunflower seed oil (10 g) was placed in a hot frying pan (33 cm diameter, 6 cm depth). When the oil was smoking (approximately 120 °C), 100 g of leafy vegetables was added to it, stir-fried for 2 min, drained, and dabbed with blotting paper for excess oil absorption.

Steaming: leafy vegetables (100 g) were placed in a dish, which was positioned on a metal bracket in a closed steamer with 500 mL of boiling tap water. The vegetables were heated over boiling water for 2 min, removed, and drained.

Microwaving: leafy vegetables (100 g) were placed in a plastic bowl, and 10 mL of tap water was poured over the vegetables. A lid was then placed on the bowl to reduce moisture evaporation, and the bowl was microwaved at 700 W for 2 min. The vegetables were removed from the bowl and drained.

Boiling: a 2 L beaker was filled with 1 L of tap water, which was brought to the boil on an electric stove. Leafy vegetables (100 g) were placed in the boiling water, cooked for 2 min, removed from the beaker, and drained.

After the cooking, the drained samples were placed in sealed plastic bowls and the weight change was determined. The sample were then mashed using a food processor and used for the parametric measurement.

### 2.3. Storage Process

Cooked vegetables were used for the storage experiments. Weight changes were recorded for vegetables stored at room temperature (28 ± 2 °C). At storage time points of 0, 12, 24, 36, and 48 h, corresponding samples were taken out and temporarily stored in a cryogenic refrigerator at −80 °C for unified measurement after the final room temperature storage time point. The nitrate, nitrite, antioxidant capacity, ascorbic acid, and total phenol at these time points were determined for all the samples.

### 2.4. Parametric Measurement

#### 2.4.1. Determination of Moisture Content

For moisture determination, 100 g of the respective samples was heated in a convection oven at 105 °C to a constant weight [17]. The moisture content of the vegetables was calculated.

#### 2.4.2. Determination of Nitrate and Nitrite

The procedure described by Ding et al. [10] was used for the detection of nitrates and nitrites but with some modifications. Briefly, 5.0 g of mashed samples was added to 25 mL of distilled water and 1.2 mL of 2.6% (weight/volume; *w/v*) Na_2_B_4_O_7_ solution, followed by heating at 70 °C for 15 min. Next, the mixture was placed in a cold water bath for 15 min, following which 0.5 mL of 30% (*w/v*) ZnSO_4_ 7H_2_O solution was added with shaking for 15 s and, subsequently, 0.5 mL of 15% (*w/v*) K_4_Fe(CN)_6_ 3H_2_O solution was added and mixed by shock. The mixture was allowed to stand for 30 min at room temperature, following which it was filtered through qualitative filter paper; the volume of the filtrate was fixed at 50 mL. The filtrate was subsequently analyzed for nitrite and nitrate content.

#### 2.4.3. Determination of Antioxidant Capacity

The antioxidant capacity was determined using 2,2-diphenylpicrylhydrazyl (DPPH) radical scavenging activity and ferric reducing antioxidant power (FRAP), using a modification of the method described by Faller and Fialho [18] and Benzie and Strain [19], respectively. Briefly, 2.0 g of mashed samples was mixed with 80% ethanol to 20 mL at a constant volume, treated with ultrasound for 10 min in an ice bath, and extracted at 4 °C for 24 h in the absence of light. The supernatant was centrifuged at 10,000 rpm for 20 min at 4 °C, and the obtained supernatant was used for the determination of antioxidant capacity.

L-ascorbic acid solution (prepared using L-ascorbic acid reagent and 80% ethanol) was used as a standard curve, as per the methods of Sdiri et al. [20] and Manthong et al. [21]. The antioxidant capacity was expressed as ascorbic acid equivalent.

#### 2.4.4. Determination of Ascorbic Acid and Total Phenol

The determination of ascorbic acid and total phenol content was based on modifications of the methods reported by Mirzaei et al. [22] and Faller and Fialho [18], respectively. 2.0 g samples were mixed with 80% ethanol to 20 mL at a constant volume, treated with ultrasound for 10 min in an ice bath, and then extracted at 4 °C for 24 h without light. The supernatant was centrifuged at 10,000 rpm for 20 min at 4 °C, and the obtained supernatant was used for the determination of ascorbic acid and total phenol. Gallic acid was used as a standard curve, and the results were expressed as gallic acid equivalent.

#### 2.4.5. Determination of Membrane Permeability

The determination of membrane permeability was based on a modification of the method described by Lu et al. [23]. Membrane permeability was expressed in terms of relative conductivity.

### 2.5. Potential Nitrite Safety Risk (PNSR) Assessment

According to Ding et al. [10], the antioxidant/in vivo nitrite ratio (A/N) was used to reflect the combined effects of cooking methods on nitrate and antioxidant properties and to evaluate the PNSR of vegetables. It was calculated as follows:

A/N = A/(*N_i_* + *N_v_*) (1)

A represents the ascorbic acid equivalent of antioxidant capacity (mmoL/kg); *N_i_*, nitrite content (mmoL/kg); and *N_v_*, in vivo nitrite levels estimated as 6.5% of the nitrate content on a molar basis.

### 2.6. Calculation Method and Statistical Analysis

To ensure the comparability of data between different cooking methods, the results of the analyses were expressed as dry weight content according to the following formula:

Composition content (DW) = C × *W_w_*/*W_d_* (2)

C represents the content of the indicated substance in the vegetables after cooking; *W_w_* the wet weight of the vegetables after cooking; and *W_d_* the dry weight of the vegetables after cooking.

All experiments were repeated four times and the data were expressed as mean ± standard deviation. Data processing was performed using the Origin statistical analysis software (version 2021; OriginLab Inc., Northampton, MA, USA). One-way analysis of variance (ANOVA), principal component analysis (PCA), and cluster analysis were performed using the IBM SPSS Statistics software (version 23.0; SPSS Inc., Chicago, IL, USA). Differences among means were considered significant at *p* < 0.05 with Duncan’s multiple range tests. PCA can consider complex patterns of variable interactions and can be combined with cluster analysis in the determination of the association of cooking methods, storage time, and various vegetable parameters with nitrite safety risk.

## 3. Results and Discussion

### 3.1. Cooking Process

#### 3.1.1. Changes in Nitrate and Nitrite

**Figure 1:**
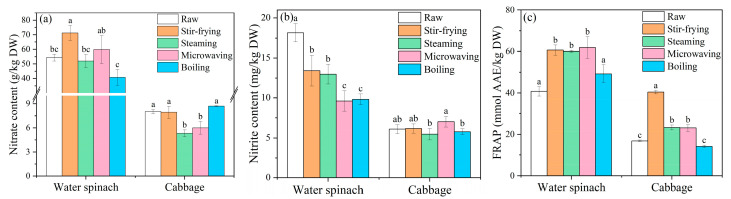
The effect of cooking process on nitrate content, nitrite content and ferric reducing antioxidant power (FRAP) in vegetable: (**a**) Nitrate; (**b**) Nitrite; (**c**) FRAP; DW: dry weight; AAE: ascorbic acid equivalent; a–c: letters a, b, c indicate values that not differ significantly from values bearing the same letter, but are significantly different to values without the same letter. (*p* < 0.05, *n* = 4).

The effect of cooking on the nitrate and nitrite content in vegetables is shown in Figure 1. The nitrate content of water spinach significantly increased by 31% after stir-frying (*p* < 0.05) but decreased by 25% after boiling. This result is similar to a previous study by Kmiecik et al. [24], who demonstrated that boiling caused a reduction in nitrate content by removing 27% and 45% of nitrates in dry matter from leaves and whole dill plants, respectively. Additionally, microwaving caused an increase in nitrate content in water spinach, but it was not significant (*p* > 0.05); steaming produced no effect on nitrate content (Figure 1a). The influence of the cooking method on the nitrate content of cabbage was clearly distinct from that of water spinach. Stir-frying and boiling had no evident effect on nitrate content, while steaming and microwaving significantly reduced the nitrate content by 33% and 25%, respectively (*p* < 0.05).

The nitrate content of water spinach increased with stir-frying (Figure 1a). Previous studies have indicated that frying increases the nitrate content of vegetables due to the loss of water during frying [25]. Chetty and Prasad [14,25] also observed an increase in nitrate contents in leafy vegetables and root vegetables after frying, and suggested that in addition to the effect of water loss, nitrogen in oil such as ammonia may also be a contributing factor. In this experiment, the nitrate content showed an increase after expressing the results as dry weight content, which suggest that there may be other reasons for increased nitrate content other than water loss. The reason for the decrease in nitrate content in water spinach during boiling is that water-soluble nitrate easily dissolves and leads to loss [25]. The variation in nitrate content in cabbage after cooking showed that boiling and stir-frying had no significant effect, while steaming and microwaving significantly reduced nitrate content; this variation was different from that in water spinach, and this difference may be related to the vegetable variety. More specifically, the disparity in the influence of cooking methods on the substance content of vegetables might be related to the differences in cellular components among different types of vegetables [26]. This theory is supported by the changes observed in the membrane permeability of vegetables after cooking, in this study (Figure S2).

The nitrite content of water spinach was significantly reduced by the four cooking methods (*p* < 0.05; Figure 1b), consistent with the results reported by Jaworska [27], which indicated that cooking reduced the nitrite content of spinach by 0–16%. Microwaving and boiling reduced the nitrite content by 47% and 46%, respectively. This reduction was greater than that induced by stir-frying and steaming. Compared with nitrate content, nitrite content in fresh water spinach is very low (Figure 1), which is easy to be quickly lost in the early stage of cooking by being lost to the environment or reduced by antioxidants. In contrast, the nitrite content of cabbage (cruciferous vegetables) did not change significantly after cooking, consistent with the findings of Leszczyńska et al. [11] who found that the level of nitrite in cruciferous vegetables did not change after cooking. However, this phenomenon may be related to the type of vegetable.

#### 3.1.2. Changes in Antioxidant Capacity

Among the two methods used in this experiment to determine the antioxidant capacity of vegetables, FRAP is considered the best for determining the antioxidant capacity of fruits and vegetables [28]. This is because the oxidation potential of DPPH radical scavenging activity is relatively low, and some antioxidant substances may not be detected compared with that by the FRAP [29]. Kim et al. [30] also detected no significant correlation between DPPH radical scavenging activity and total phenol content (R = 0.2565). Thus, the antioxidant capacity reflected by DPPH radical scavenging activity is lower than that of FRAP. Therefore, in the subsequent calculation of antioxidant/in vivo nitrite ratio (A/N), the FRAP of leafy vegetables was used to represent the antioxidant capacity of vegetables (DPPH radical scavenging activity is displayed in Figure S3 for reference).

The effect of cooking on the FRAP of the vegetables is shown in Figure 1c. Different cooking processes had the same effect on the FRAP of the two leafy vegetables. Stir-frying, steaming, and microwaving significantly increased the FRAP of the vegetables (*p* < 0.05), while boiling produced no obvious effect (*p* > 0.05). For water spinach, stir-frying, steaming, and microwaving increased the FRAP by 49%, 48%, and 51%, respectively, producing similar effects. However, for cabbage, stir-frying increased the FRAP by 140%. This increase is considerably higher than that observed for by steaming (38%) and microwaving (37%).

In general, less stable antioxidants, such as ascorbic acid and total phenol, in vegetables are destroyed by heat, resulting in a decrease in the antioxidant capacity of vegetables, which is inconsistent with the results of the present study. Previous studies have reported a similar phenomenon to this study; the total phenolic or carotenoid content and the total antioxidant capacity of vegetables increased after frying [31,32]. Ali [33] showed that vitamin C content in cauliflower was only slightly affected during frying. On the other hand, Ramírez-Anaya et al. [34] found that when frying with certain oils such as extra virgin olive oil (EVOO), the antioxidant capacity of vegetables increased due to the transfer of phenols from EVOO to vegetables during cooking. But in this study, the antioxidant capacity of sunflower oil used in stir-frying is negligible (Table 1), suggesting that sunflower oil does not directly provide additional antioxidants to vegetables. Therefore, the increase in antioxidant capacity of stir-fried cabbage may be related to new substances produced during cooking. Pérez-Burillo et al. [35] demonstrated that Maillard reaction parameters (furoline and 5-hydroxymethylfurfural) were positively correlated (*p* < 0.05) with the total antioxidant capacity of vegetables. Miglio et al. [36] and Ashour and El-hamzy [37] indicated that the increase in antioxidant capacity during frying could be attributed to the formation of new molecules with high antioxidant capacity, such as Maillard reaction products.

Previous studies have shown that steaming increases the total phenol content and antioxidant capacity of vegetables [38], which is consistent with the results of the present study. Wachtel-Galor et al. [16] suggested that steaming is a good way to release or preserve the antioxidant properties of vegetables, because the antioxidant capacity of all vegetables used in their study increased after steaming and were least affected by the cooking time. The effects of microwaving were considered inconclusive in previous studies [12]; most of them were attributed to differences in vegetable varieties.

During boiling, water-soluble antioxidants can leach into the wastewater, causing loss of these antioxidants [38]. Unlike the other three cooking methods, boiling was considered to have no significant effect on the antioxidant capacity of vegetables in the present study, which is inconsistent with previous reports [39]. This difference may be attributed to the cooking time. Ali [33] demonstrated that the antioxidant capacity of cauliflower decreased with an increase in cooking time. However, the boiling time in the present study (2 min) was shorter than that in previous studies (4 min or longer), thus reducing the loss in antioxidant capacity in the vegetables.

#### 3.1.3. Assessment of PNSR of Cooked Vegetables

**Figure 2:**
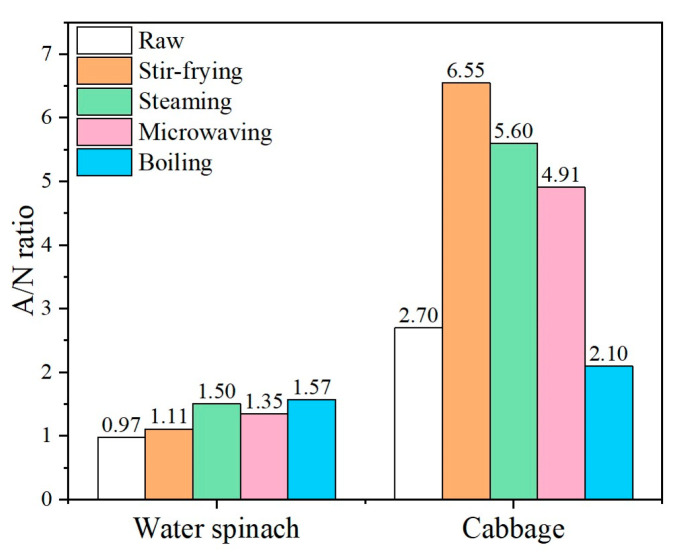
The antioxidant/in vivo nitrite ratio (A/N) of cooked vegetables.

The influence of cooking methods on the chemical substances in leafy vegetables is a complex process. However, according to Ding et al. [10], the comprehensive influence of cooking methods on nitrate and antioxidant capacity in cooked vegetables can be directly reflected by the antioxidant/in vivo nitrite ratio (Figure 2). The essence of the A/N ratio is the equilibrium of antioxidant capacity and nitrate content, which is used to evaluate the PNSR of vegetables. The higher the ratio, the lower the PNSR associated with the cooking process. It should be noted that the A/N ratio has a limitation. It was used to compare relative changes in the potential nitrite risk associated with various cooking methods. And the nitrite safety risk evaluated by the A/N ratio is potential, mainly because of the in vivo nitrite that is converted from excessive intake of nitrate. From Figure 2, the highest A/N ratio for cooked water spinach was observed with boiling (1.57), whereas that for cooked cabbage was observed with stir-frying (6.55).

All four cooking methods improved the A/N ratio for water spinach relative to the ratio of the raw state. However, their degree of improvement was different (Figure 2). Water spinach has a nitrate content of up to 54.4 g/kg dry weight (Table 1). Due to the higher initial nitrate content of water spinach, boiling achieves the highest A/N ratio by reducing the amount of nitrate content, even though it does not significantly increase antioxidant capacity compared to the other three methods (Figure 1a). Steaming is also a potential cooking method for water spinach, which achieved a high A/N ratio (1.50) by increasing antioxidant capacity but did not affect the nitrate content of water spinach. However, it produced a slightly lower A/N ratio than boiling in this study (Figure 2). Stir-frying increased the antioxidant capacity of cabbage and thus achieved the highest A/N ratio, while the A/N ratio with boiling was lower than that for the raw state because of the negative effect of boiling on antioxidant capacity (Figure 1c). Additionally, steaming and microwaving increased the A/N ratio of cabbage relative to the raw state by reducing the nitrate content while increasing antioxidant capacity (Figure 1). However, this increase was not more than that with stir-frying. Therefore, compared with the other cooking methods, boiling is considered a safer method for cooking water spinach and stir-frying is more suitable for cabbage, according to the A/N ratio.

### 3.2. Storage Process

#### 3.2.1. Changes in Nitrate and Nitrite

According to the A/N ratio (Figure 2), boiling and stir-frying were evaluated as suitable cooking methods for water spinach and cabbage, respectively. Therefore, we further evaluated the parameter changes for boiled water spinach and stir-fried cabbage during storage to explore the actual nitrite risk in vegetables stored overnight.

**Figure 3:**
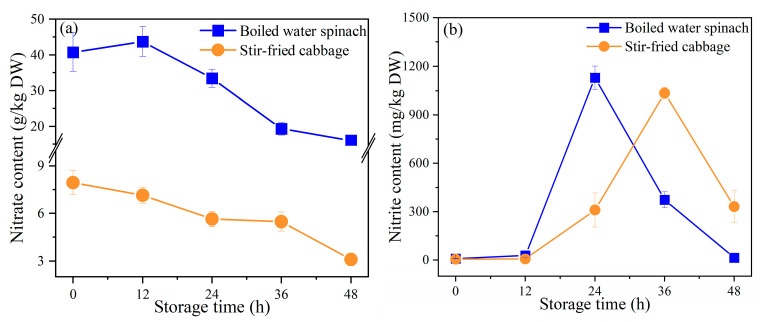
Content of nitrate and nitrite in leafy vegetables during storage: (**a**) nitrate content; (**b**) nitrite content; DW, dry weight.

The nitrate and nitrite contents in leafy vegetables during storage are shown in Figure 3. The nitrate content in vegetables continued to decrease during storage (Figure 3a), while the nitrite content in vegetables increased rapidly to a peak at first and then decreased gradually. There was almost no change in nitrite content during 0 to 12 h; however, it was significantly increased to 1130.0 mg/kg dry weight between 12 to 24 h for boiled water spinach and significantly increased to 1035.8 mg/kg dry weight from 12 to 36 h in cabbage (Figure 3b). In addition, the nitrite content of boiled water spinach and stir-fried cabbage peaked at 24 h and 36 h, respectively, but subsequently began to decrease gradually, decreasing by 99% and 68%, respectively, at 48 h (Figure 3b).

The decrease in nitrate content and the rapid increase in nitrite content during 12–24 h was due to the effect of microorganisms in the storage environment. Previous studies have found that nitrate in vegetables can be reduced to nitrite by microorganisms (nitrate reductase) during storage at room temperature, resulting in a significant increase in nitrite content and a decrease in nitrate content [40]. The decrease in nitrite content at the later stage of the storage process might be because the rate of microbial consumption of nitrite exceeded the rate of nitrate reduction. Chen et al. [41] studied the biochemical reaction pathways and related enzyme activities of microorganisms during the fermentation of Paocai, which is similar to the role of microorganisms observed during storage in this study; they believed that the nitrite in Paocai can be converted to nitrogen (N_2_) by denitrification and to ammonia (NH_4_^+^) by nitrate reduction, leading to a decrease in nitrite content.

#### 3.2.2. Changes in Antioxidant Capacity and Key Antioxidants Content

**Figure 4:**
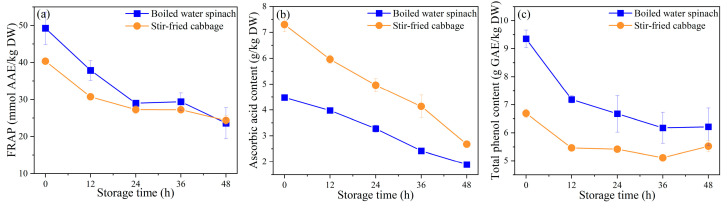
Ferric reducing antioxidant power (FRAP) and key antioxidants content of leafy vegetables during storage: (**a**) FRAP; (**b**) ascorbic acid content; (**c**) total phenols content; DW, dry weight; AAE, ascorbic acid equivalent; GAE, gallic acid equivalent.

The antioxidant capacity (FRAP) and contents of key antioxidants in leafy vegetables during storage are presented in Figure 4, and the DPPH radical scavenging activity are displayed in Figure S4 for reference. After 48 h of storage, only 48% and 60% of antioxidant capacity was retained in boiled water spinach and stir-fried cabbage, respectively. Most of the antioxidant capacity loss occurred in the first 24 h of storage, accounting for 79% and 81% of the total loss, respectively (Figure 4a).

Ascorbic acid and total phenols are the main sources of the antioxidant capacity of vegetables. Ascorbic acid in boiled water spinach and stir-fried cabbage decreased by 58% and 63% after 48 h of storage, respectively (Figure 4b). Ascorbic acid is highly sensitive to cooking and storage and is easily oxidized during storage, resulting in a large loss [42]. Balan et al. [43] reported similar results; spinach and broccoli lost 81.23% and 62.66% ascorbic acid after seven days of cold storage, respectively, and most of the loss occurred during early storage. The total phenolic content of boiled water spinach and stir-fried cabbage decreased by 29% and 19% during the early storage period (0–24 h) and subsequently tended to be stable (Figure 4c). On the basis of a previous report, changes in phenolic compounds are believed to be related to various abiotic or biological stresses on vegetables [44]. However, in this study, most of the physiological responses of the vegetables themselves stopped after cooking due to enzyme inactivation; therefore, changes in total phenolic content during storage may be related to oxidation or microorganisms.

#### 3.2.3. Principal Component Analysis (PCA)

**Figure 5:**
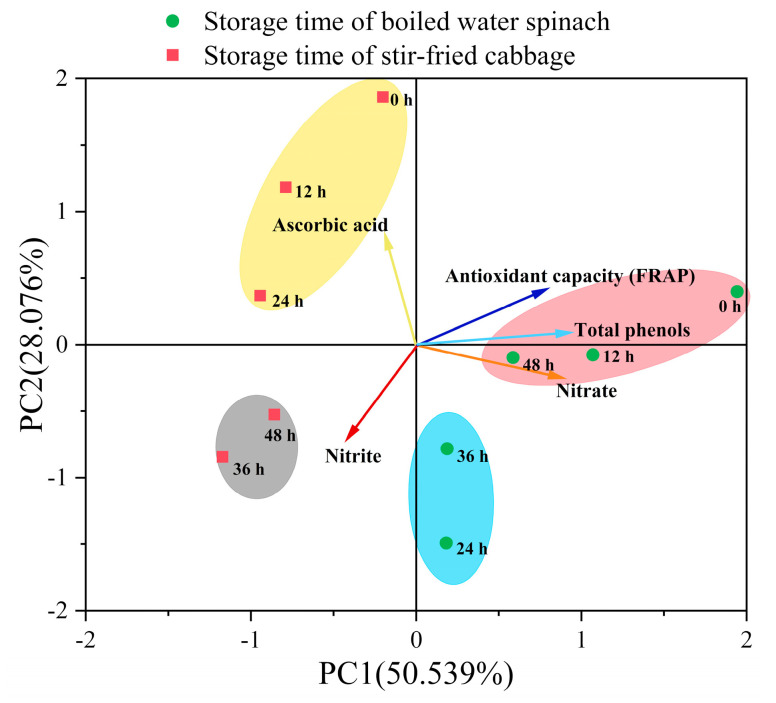
Principal component analysis (PCA) and clustering analysis of the storage process of leafy vegetables: the circles in different colors represent the samples classified to different types.

The PCA of the correlation between the parameters of cooked vegetables during the storage process is shown in Figure 5. PC1 was primarily related to the indirect risk of nitrite and was associated with parameters such as nitrate content, antioxidant capacity, and total phenol content. PC2 was primarily related to the direct risk of nitrite and was associated with parameters such as nitrite and ascorbic acid contents.

PCA showed that the indirect risk of nitrite (PC1) decreased while the direct risk of nitrite (PC2) increased during the storage of boiled water spinach. Among the storage periods, 0 and 12 h of storage indicated high indirect risk but low direct risk, and storage periods of 24 and 36 h indicated low indirect risk but high direct risk. After 48 h of storage, boiled water spinach had already exhibited a nitrite peak, and from sight and smell observation was apparently spoiled even though the direct risk was low. For stir-fried cabbage, the indirect risk was slightly reduced during storage, while the direct risk increased substantially. Among them, the direct risk is low when stored for 0, 12, and 24 h but high when stored for 36 and 48 h. Therefore, considering the direct nitrite risk, the storage time of boiled water spinach should not exceed 12 h, and that of stir-fried cabbage should not exceed 24 h.

In addition, the indirect risk of nitrite in water spinach was always higher than that in cabbage during storage (PC1), which was determined by the vegetable species. According to Figure 3, nitrite formation concentration of the two leafy vegetables was similar in the storage process. However, the content of nitrate was much higher than that of nitrite formation concentration. Moreover, the nitrate content of water spinach was much higher than that of cabbage, indicating that indirect risk was highly correlated with the type of vegetables. These results indicate that some leafy vegetables, as represented by water spinach, which are prone to nitrate accumulation, were more likely to produce PNSR during eating and storage. Therefore, people should choose appropriate cooking methods and storage periods to reduce the PRSR of these vegetables.

## 4. Conclusions

This study investigated the effects of four common domestic cooking methods on the equilibrium of antioxidant capacity and nitrate content in water spinach and cabbage. The potential nitrite safety risk (PNSR) of cooked vegetables on the basis of the A/N ratio was also evaluated for the first time. Water spinach contains a large amount of nitrate. Therefore, boiling achieved the highest A/N ratio by reducing the nitrate content, on account of which it was considered a safer method for cooking water spinach than the other methods. Stir-frying was found to be more suitable for cabbage in this study, as it significantly increased the antioxidant capacity and achieved the highest A/N ratio. Obviously, the differences in suitable cooking methods for leafy vegetables are related to the species. They may be attributed to the nature of the vegetables themselves, such as the nitrate accumulation capacity and differences in the physical structure of the vegetable cells. These differences result in different degrees of damage after cooking (heat treatment), which requires further study. In addition, the cooking time is worthy of further study in the future, because the substances in vegetables will change with the extension of cooking time, resulting in further changes in the PNSR of vegetable.

Eating vegetables stored overnight is inevitable for many people. Storage experiments showed that the storage time of boiled water spinach and stir-fried cabbage should not exceed 12 h and 24 h at room temperature, respectively; otherwise, there is a direct nitrite safety risk. Furthermore, leafy vegetables prone to excessive accumulation of nitrate, such as water spinach, have a high PNSR during eating and storage. These vegetables should be treated carefully using appropriate cooking methods and limited storage time to prevent adverse health effects.

## Figures and Tables

**Table 1:** Characteristics of raw materials used in the experiment.

Vegetable	Nitrate Content (g/kg DW) ^a^	Nitrite Content (mg/kg DW) ^a^	Antioxidant Capacity (FRAP) ^d^ (mmoL AAE/kg DW) ^a,b^	Moisture Content (%)
Water spinach	54.4 ± 2.4	18.2 ± 1.1	40.7 ± 2.3	94.2 ± 1.5
Cabbage	8.0 ± 0.3	6.1 ± 0.6	16.8 ± 0.4	93.4 ± 0.3
Sunflower seed oil ^c^	ND	ND	<0.1	-

^a^ DW, dry weight. ^b^ AAE, ascorbic acid equivalent. ^c^ ND, not detected. ^d^ FRAP, ferric reducing antioxidant power.

## Data Availability

The data presented in this study are available on request from the corresponding author. The data are not publicly available due to privacy.

## Supplementary Materials

The following are available online at https://www.mdpi.com/article/10.3390/foods10122953/s1. Figure S1: Apparent state of leafy vegetables after cooking for different time; Figure S2: Membrane permeability of leafy vegetables after cooking; Figure S3: The effect of cooking process on DPPH radical scavenging activity of vegetables; Figure S4: DPPH radical scavenging activity of leafy vegetables during storage.
