# Supplementary material for: Assessment of Potential Nitrite Safety Risk of Leafy Vegetables after Domestic Cooking
Source: Foods. 2021 Dec 1;10(12):2953. doi: 10.3390/foods10122953 (PMC8701323; doi:10.3390/foods10122953)
Supplement: Supplementary file 1 [file foods-10-02953-s001.zip › foods-1465873-supplementary.pdf]

Supplementary materials

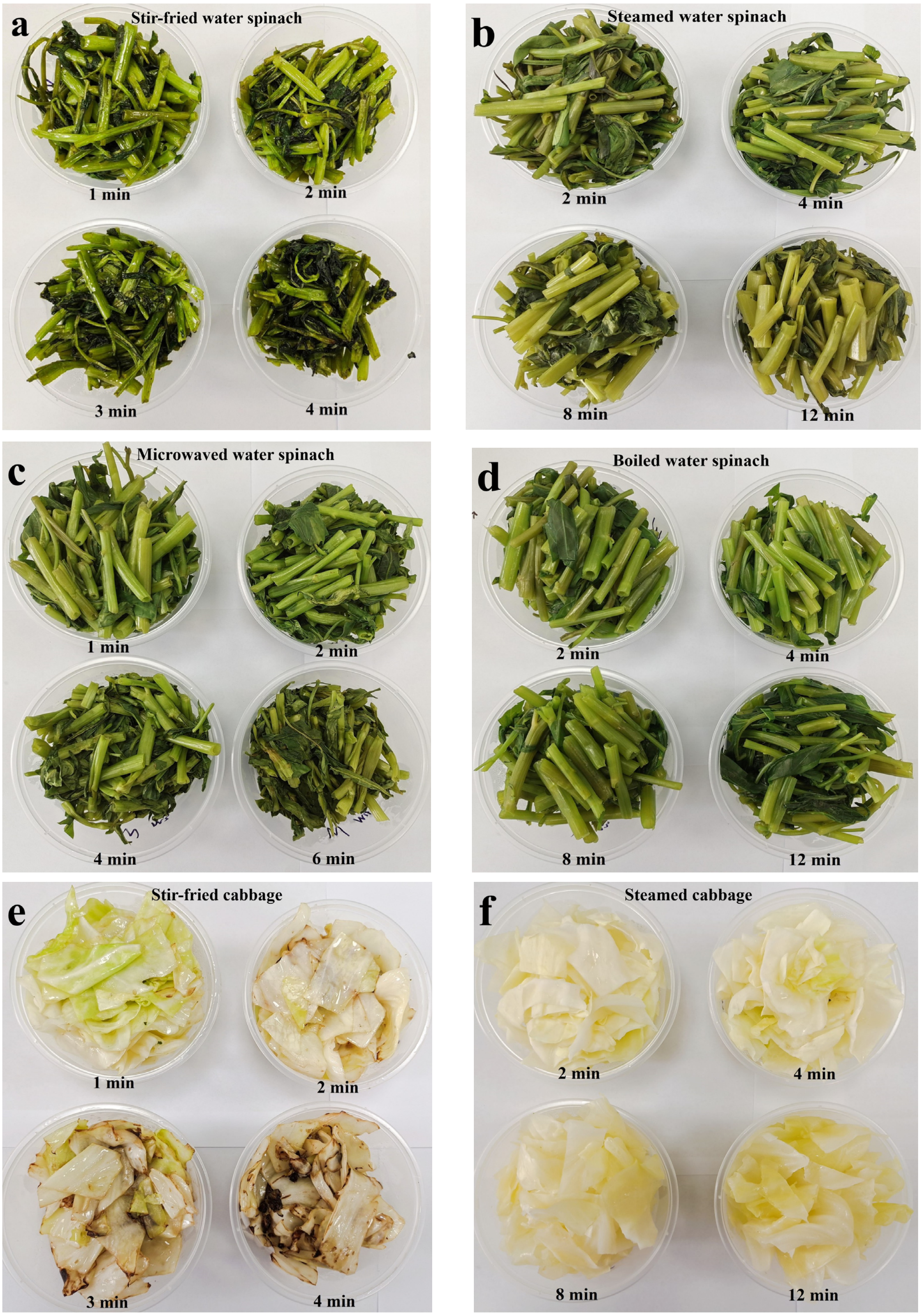

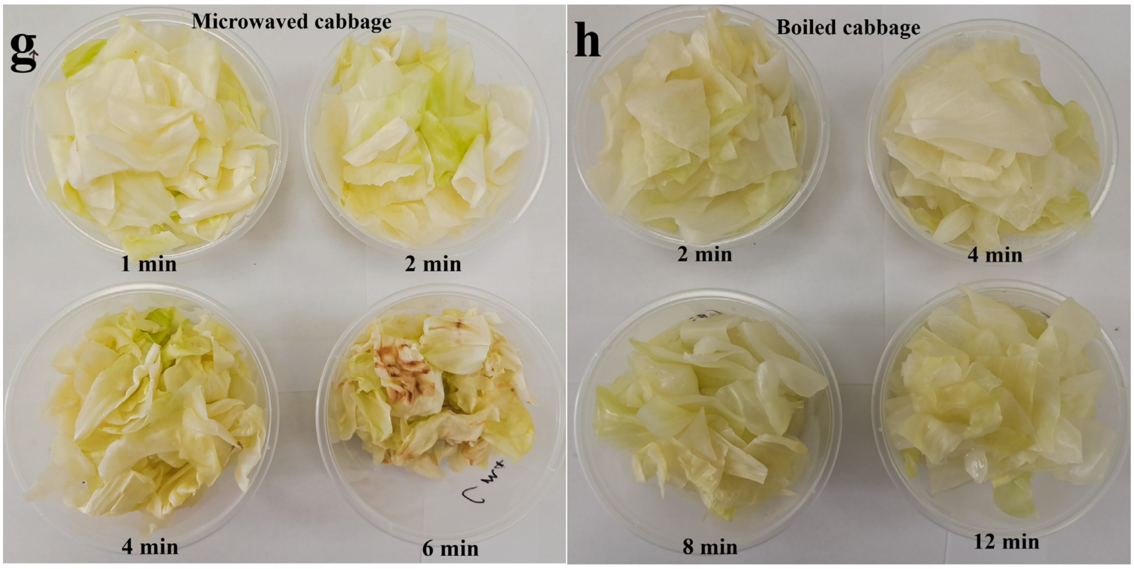

Figure S1: Apparent state of leafy vegetables after cooking for different time. (a): Stir-fried water spinach; (b): Steamed water spinach; (c): Microwaved water spinach; (d): Boilied water spinach; (e): Stir-fried cabbage; (f): Steamed cabbage; (g): Microwaved cabbage; (h): Boilied cabbage.

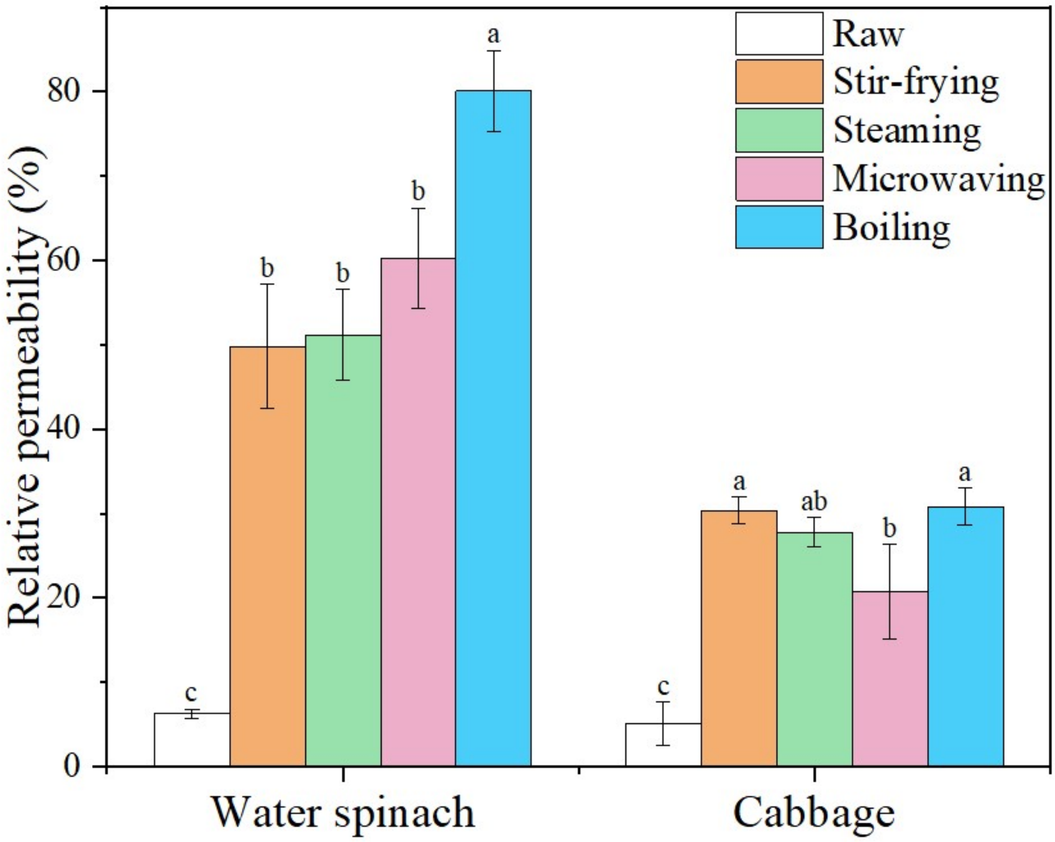

Figure S2: Membrane permeability of leafy vegetables after cooking. a–c: letters a, b, c indicate values that not differ significantly from values bearing the same letter, but are significantly different to values without the same letter. ($p < 0.05$, $n = 4$)

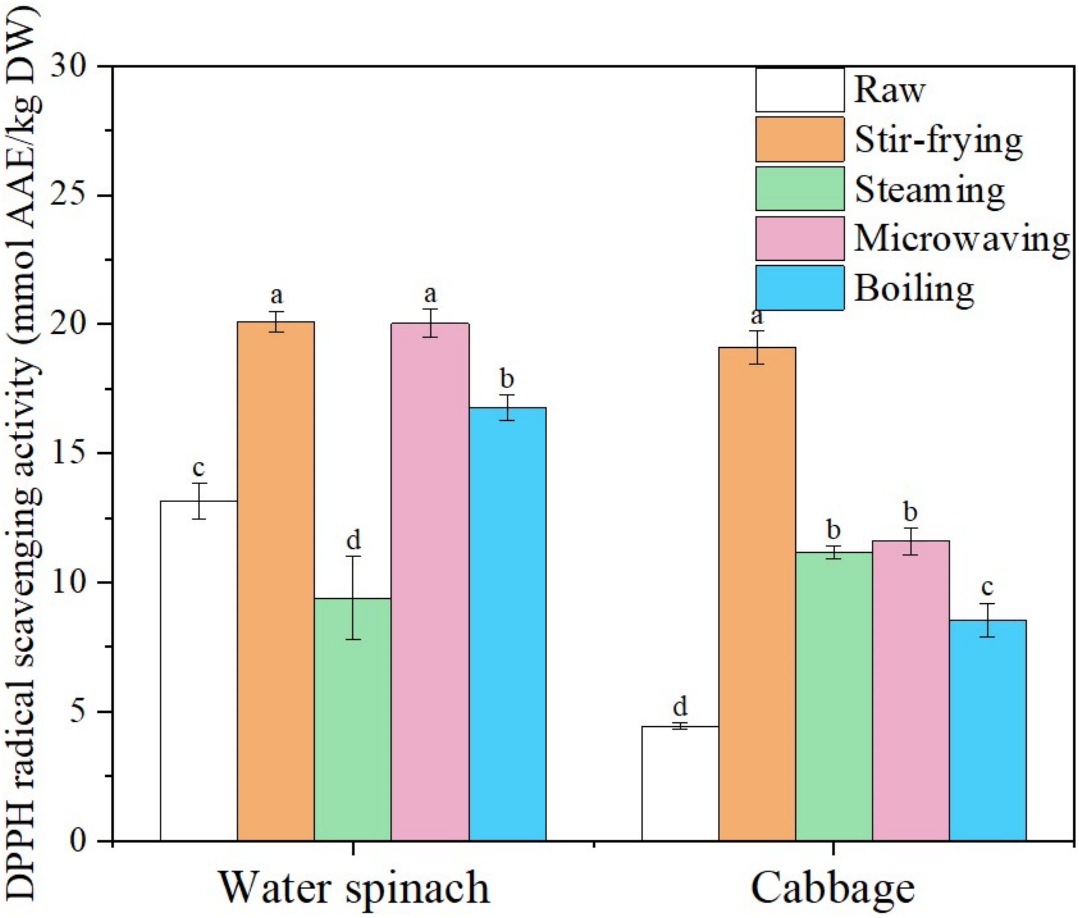

Figure S3: The effect of cooking process on DPPH radical scavenging activity of vegetables. a–d: letters a, b, c, d indicate values that not differ significantly from values bearing the same letter, but are significantly different to values without the same letter. ($p < 0.05$, $n = 4$)

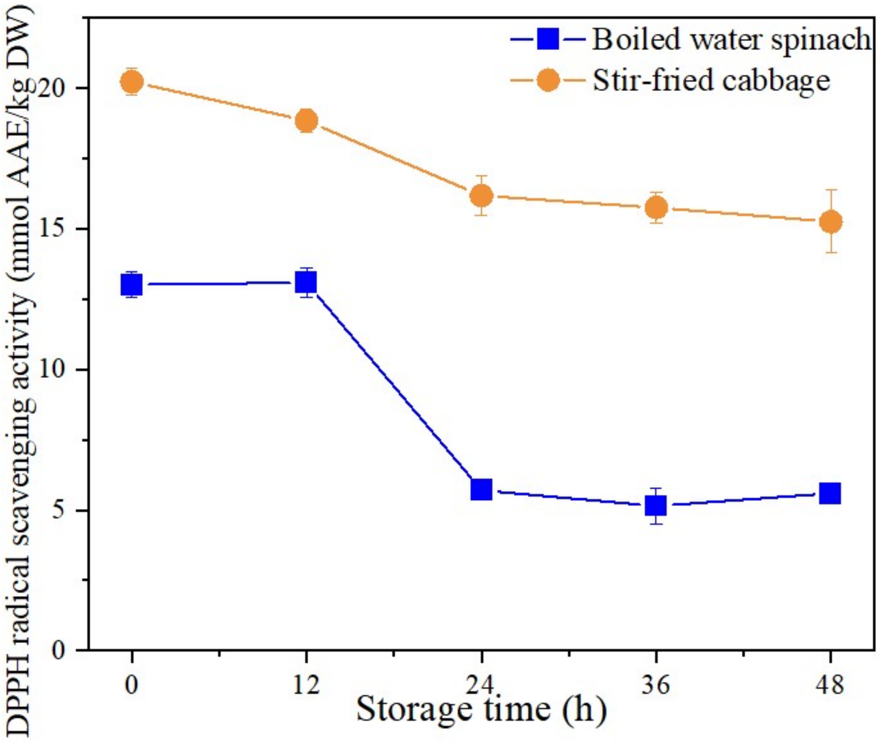

Figure S4: DPPH radical scavenging activity of leafy vegetables during storage.
